# Non-Invasive Analysis of Human Liver Metabolism by Magnetic Resonance Spectroscopy

**DOI:** 10.3390/metabo11110751

**Published:** 2021-10-29

**Authors:** John G. Jones

**Affiliations:** CNC—Center for Neuroscience and Cell Biology, CIBB—Centre for Innovative Biomedicine and Biotechnology, University of Coimbra, 3004-504 Coimbra, Portugal; john.griffith.jones@gmail.com; Tel.: +351-231-249-181

**Keywords:** in vivo magnetic resonance, liver metabolism, hyperpolarization, stable isotopes

## Abstract

The liver is a key node of whole-body nutrient and fuel metabolism and is also the principal site for detoxification of xenobiotic compounds. As such, hepatic metabolite concentrations and/or turnover rates inform on the status of both hepatic and systemic metabolic diseases as well as the disposition of medications. As a tool to better understand liver metabolism in these settings, in vivo magnetic resonance spectroscopy (MRS) offers a non-invasive means of monitoring hepatic metabolic activity in real time both by direct observation of concentrations and dynamics of specific metabolites as well as by observation of their enrichment by stable isotope tracers. This review summarizes the applications and advances in human liver metabolic studies by in vivo MRS over the past 35 years and discusses future directions and opportunities that will be opened by the development of ultra-high field MR systems and by hyperpolarized stable isotope tracers.

## 1. Introduction

The liver represents a key metabolic node in the body encompassing nutrient transformation and fuel homeostasis as well as detoxification of ethanol and xenobiotic compounds. Its relatively large size and body location, coupled with a dynamic metabolome that features high concentrations of a diversity of metabolites such as glycogen, glutamine, ATP, sugar phosphates, phosphocholine and phosphoethanolamine, has made it an attractive target for in vivo magnetic resonance spectroscopy (MRS) studies of hepatic metabolism since the early days of in vivo MRS development [1,2,3,4]. Given that many diseases cause substantial changes in hepatic intermediary metabolism coupled with the availability of higher-field MRS systems for both in vivo human and animal model studies, there is high and ongoing interest in applying this methodology to further our understanding of hepatic intermediary metabolism in physiological and pathophysiological settings. The purpose of this review is to highlight the versatility of multinuclear in vivo MRS both in direct observation of hepatic metabolites as well as hepatic metabolite enrichment from metabolic stable-isotope tracers.

### 1.1. Observation of Hepatic Metabolites by MRS

There are several aspects that increase the difficulty of performing in vivo MRS spectroscopy of the liver compared to other large organs such as the brain. These have been previously discussed in detail [5] and can be summarized as follows: First, there is considerable inter-individual variability in its gross structure (i.e., configuration of the lobes and major vessels, therefore the region for observation must be carefully tailored for each individual with particular attention to exclude extra-hepatic tissues such as muscle or adipose tissue. Second, in a resting supine individual, the liver position is not static. This is primarily due to diaphragm movement during breathing but other involuntary processes such as intestinal peristalsis and pulsatile blood flow also contribute. This affects ^1^H signals in particular hence ^1^H MRS data are typically acquired periodically while the subject holds their breath [5,6,7,8]. Thirdly, the liver has higher levels of iron compared to many other tissues which results in paramagnetic broadening of MR signals.

### 1.2. Advances in MRS Instrumentation

Since the development of whole-body MR scanners with fields of 0.5–1.5 T in the 1980’s, there has been a constant push for systems with ever higher magnetic fields. Currently, 3 T systems are becoming widespread and in 2017, the Food and Drugs Administration approved a 7 T system as a magnetic resonance imaging device. As of now (2021), the highest operating field for human subjects is 10.5 T at the University of Minnesota facility, and initial studies indicate that subject safety is not compromised in this setting [9]. There are ongoing efforts to develop systems of 11.7 T (AROMA consortium H2020 grant agreement No 885876) and initiatives for the development of 14–20 T systems [10]. For in vivo MRS spectroscopy, higher magnetic fields deliver an increase in signal dispersion that scales directly with the increase in the applied field (B_0_) while the signal-to-noise ratio (SNR) increases as B_0_^1.65^ [11]. At the same time, there is also the need for increased radiofrequency (RF) power deposition that may exceed safety limits. This is primarily an issue for broad-band decoupling of high-gamma nuclei such as ^1^H. In addition, establishing field homogeneity and operating imaging gradients for localized spectroscopy is more challenging at higher fields. Among other things, this increases the difficulty of obtaining narrow MR signals from deeper hepatic regions. Finally, both spin-lattice (T_1_) and transverse relaxation times (T_2_) of many metabolites are sensitive to magnetic field strength [12,13] which can compromise the efficacy of signal collection and alter the relationship between signal intensity and metabolite concentration. To date, there is a strong consensus that the advantages of higher fields far outweigh these drawbacks, particularly for MRS with low-gamma nuclei [10,11,14].

Since high field MRS has been primarily driven by studies on the brain, the integral RF transmit/receive components of high field instruments are optimized for the head rather than the abdominal region. Therefore, liver MRS studies with these systems have required the development of bespoke RF coils and antenna systems [15,16,17].

### 1.3. In Vivo ^1^H MRS of Liver

^1^H is the default observation nucleus for clinical imaging and ^1^H body coils are also a standard feature for clinical 1.5–3.0 T MR systems. Thus, it is usually feasible to acquire localized ^1^H spectra of liver tissue on a standard hospital MR scanner.

#### 1.3.1. ^1^H MRS of Liver Lipids

To date, the most widespread application, and perhaps among the most important in terms of current clinical relevance, is the quantification of liver triglycerides. ^1^H MRS provides a precise measurement of liver triglyceride levels, with better sensitivity and specificity than other noninvasive probes of liver fat such as ultrasound [18]. This approach was initially validated in a large population (2349 participants) and established the now widely accepted threshold of 55.56 mg/g liver triglycerides concentration for non-alcoholic fatty liver disease (NAFLD), based on triglycerides concentrations measured for the 95th percentile of this study cohort [19]. More recently, the detection of the triglyceride signal has been translated into an imaging modality (magnetic resonance imaging-proton density fat fraction, MRI-PDFF) that provides information on the whole liver combined with simpler post-acquisition processing and representation of the data [20]. MRI-PDFF is now considered as the gold standard for hepatic lipid quantification in various settings [21,22]. At fields of 3 T and above, signals from mono- and polyunsaturated fatty acids become resolved allowing the abundance of these species to be measured thereby providing a lipidomic profile in addition to total liver triglycerides levels [23,24].

#### 1.3.2. ^1^H MRS of Other Hepatic Metabolites

Aside from triglycerides, other hepatic metabolites that have been quantified by ^1^H MRS include choline-containing compounds and glycogen [25,26,27] which were measured with a 3 T instrument. While many tumors have high levels of choline, in vivo ^1^H MRS measurements of hepatic choline in patients with liver tumors did not observe any significant increases in choline compared to healthy subjects [27]. Glycogen observation by conventional in vivo ^1^H MRS is hampered by several factors including short T_2_ of its hydrogens and a significant loss of signal during presaturation of the water signal due to saturation transfer [28]. By applying this process in reverse, i.e., pre-saturating the glycogen signals and observing the resulting decrease in the water signal intensity, Zhou et al. were able to follow dynamic changes in hepatic glycogen levels induced by glucagon and fasting re-feeding [29]. Since this approach only requires the quantification of the water signal, it can also be easily translated into an imaging mode.

### 1.4. In Vivo ^31^P MRS of Liver

**Figure 1:**
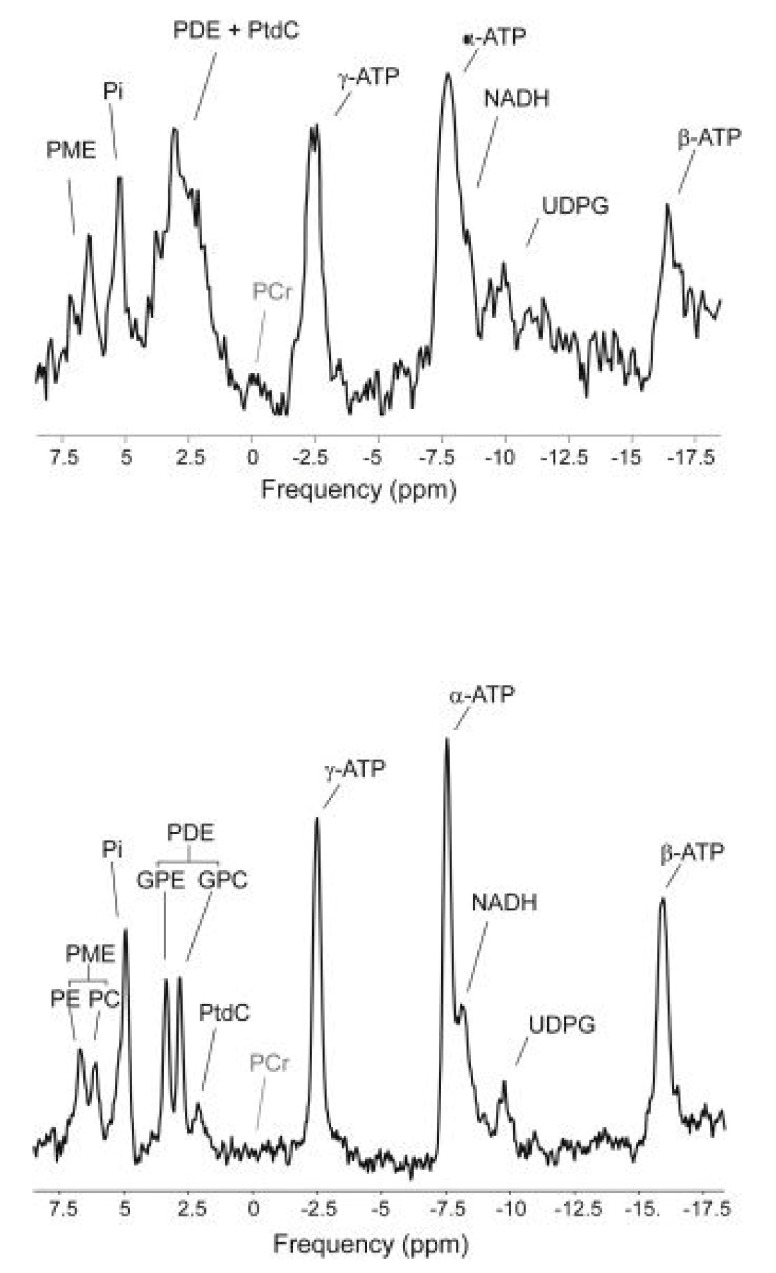
Comparison of in vivo ^31^P MR spectra of human liver acquired at 3 T (**top**) and at 7 T (**bottom**). This figure was adapted from ref. [39].

^31^P is the sole stable isotope of phosphorus with a nuclear spin of ½ and a relatively strong gyromagnetic ratio (40.5% that of ^1^H). Although the ^31^P chemical shift dispersion is much greater than that of ^1^H (~350 ppm versus ~10 ppm), metabolites of phosphorus all resonate within a 25 ppm spectral region, with phosphate mono- and diester species crowded into a ~5 ppm window. The chemical shift of inorganic phosphate (P_i_) as well as those of phosphate esters are also sensitive to pH [30,31] while those of phosphoanhydrides are influenced by the binding of various metal ions such as magnesium [32]. Saturation transfer experiments allow the transfer of phosphorus from one metabolic intermediate to another to be followed thereby providing information on rates of synthesis such as that of ATP from ADP and P_i_ [33,34,35,36]. Therefore, in addition to assaying key phosphometabolites with good precision and accuracy [37], in vivo ^31^P MRS also informs on bioenergetic status and ionic homeostasis. The ratio of phosphodiesters to phosphomonoesters signals (PDE/PME) is linked to cell membrane turnover [38,39]. PDE/PME of cirrhotic and of cancerous liver tissues were shown to differ significantly from that of a healthy liver [40,41,42]. While high resolution ^31^P NMR of liver extracts can identify over 50 different phospho-metabolites [43], the number of metabolites that can be resolved and quantified by in vivo ^31^P MRS is far less [44] but is nevertheless more diverse in comparison to that provided by ^1^H MRS. At high magnetic fields (≥7 T), the increased signal dispersion allows more hepatic phospho-metabolites to be resolved and quantified, [45,46] as exemplified by Figure 1 [46].

The challenges and potential limiting factors of high-field in vivo ^31^P NMR MRS include design and implementation of RF hardware for optimal observation of hepatic metabolites, avoidance of confounding signals from non-hepatic tissues in intimate contact with the liver such as phosphatidylcholine from the gall-bladder and phosphcreatine from surrounding muscle [4,47,48]; and maintaining efficient ^1^H-decoupling without exceeding the safe limits for tissue RF power deposition. Finally, there are hepatic studies that integrate the observation of ^31^P and ^1^H thereby providing correlated information of phospho-metabolites with other species such as lipids [49,50,51]. A portfolio of in vivo ^31^P MRS studies of human liver is shown in Table 1. This is not meant to include all reported studies to date, but instead to highlight the diversity of topics in hepatic physiology and intermediary metabolism that have been studied.

### 1.5. In Vivo ^13^C MRS of Liver

^13^C is the stable isotope of carbon with a spin of ½ and a natural abundance of 1.1%. Its gyromagnetic ratio is ~¼ that of ^1^H, therefore its overall sensitivity is several orders of magnitude less than that of ^1^H. Nevertheless, for liver metabolites that can reach high concentrations, such as glycogen and lipids, their natural abundance ^13^C signals can be observed with reasonable collection times [2,3,58]. In addition, ^13^C signals from isotopically enriched substrates and their metabolic products, where ^13^C abundance can be boosted to nearly 100-fold over background levels, can be detected [59,60,61,62,63].

The ^13^C chemical shift dispersion is much greater in comparison to ^1^H, therefore in principle it provides increased resolution of metabolites. On the other hand, the majority of metabolite carbons are bound to one or more hydrogens that result in the ^13^C signal being split by ^1^H-^13^C scalar coupling. Not only does this effectively reduce the signal-to-noise ratio by at least a factor of two, it also multiplies the number of metabolite signals within the same spectral region thereby compromising signal resolution. These effects can be eliminated by broadband ^1^H-decoupling which also provides an additional boost to the ^1^H-decopuled ^13^C singlet signal by the nuclear Overhauser enhancement (nOe) effect. As magnetic fields increase, the ^1^H-frequency decoupling bandwidth also needs to be increased resulting in higher deposition of RF power into tissues. Moreover, nOe can vary substantially between ^13^C in different molecular sites, and this must be taken into account when relating ^13^C signal intensities to absolute metabolite concentrations. Finally, the T_1_ of non-protonated carbons such as carboxyls and quaternary carbons are relatively long, which in combination with an absence of nOe, can constrain the acquisition of their ^13^C signals over short intervals. In vivo ^13^C MR of hepatic metabolism in humans is being driven forward by several innovations that directly confront the limitations described earlier. To minimize the deposition of RF power into the region of observation as a result of broadband ^1^H decoupling, bespoke decoupling schemes have been developed [64]. For studies that focus on observation of a single metabolite ^13^C signal, such as the carbon 1 resonance of glycogen, it is only necessary to decouple the hydrogen attached to this carbon, hence the decoupling bandwidth—and, therefore the power deposition—can be substantially reduced. This approach was used in one of the pioneering in vivo ^13^C MR studies of liver metabolism, which documented the decrease in the natural-abundance ^13^C1 signal of liver glycogen during fasting in healthy humans [3]. Hepatic glycogen synthesis and degradation fluxes are key components of systemic glucose homeostasis and are highly sensitive to the insulin/glucagon ratio. The application of in vivo ^13^C MR to measure changes in hepatic glycogen during fasting and feeding has advanced our understanding of hepatic carbohydrate metabolism in subjects with insulin resistance, as well as in patients with diabetes [65,66,67,68,69]. Among other things, it revealed that while both hepatic glycogen deposition and degradation rates are significantly reduced in Type-1 diabetic subjects undergoing standard insulin therapy, these fluxes may be restored to normal with intensive insulin therapy [70,71]. Acute induction of hepatic insulin resistance via infusion of lipid was shown to modify rates of hepatic glycogenolysis in the fasted state [68,72].

In some cases, ^13^C-signals of ^13^C-enriched hepatic metabolites can be resolved and quantified in the absence of ^1^H-decoupling. For example, the appearance of ingested [1-^13^C]glucose in the liver and its conversion to [1-^13^C]glycogen was observed at 3 T with 0.5 min time resolution without deployment of ^1^H decoupling [59]. In a study of hepatic Krebs cycle metabolism with [1-^13^C]acetate, ^13^C appearance in the two carboxyls of glutamate was monitored [62]. Since ^13^C nuclei in these sites have no directly attached protons, optimal observation of their ^13^C signals is not dependent on ^1^H decoupling.

The interaction of ^13^C and ^1^H via scalar coupling provides the basis for monitoring ^13^C-enrichment indirectly via observation of the attached proton(s). While ^1^H observation delivers vastly increased signal sensitivity and is also the default nucleus for in vivo localized spectroscopy with whole-body MR systems, the pulse sequences for selecting the ^1^H-^13^C-coupled signals while filtering out those from ^1^H-^12^C are more complex and require precise calibration of the RF electronics. Although, in principle, the ^1^H-^13^C-coupled signals can be resolved along both ^1^H and ^13^C dimensions, for in vivo studies, time and instrument constraints limit the signal acquisition to the ^1^H dimension only. Thus, the signal dispersion is limited to that of ^1^H, which effectively precludes shotgun observation of arrays of ^13^C-enriched metabolites but may, nevertheless be effective for observation of near-isochronous ^13^C-enriched signals such as the methylene carbons of triglyceride fatty acids [73]. Veeraiah et al. applied a similar approach to demonstrate that the background ^13^C-methylene signals of hepatic fatty acids in healthy subjects could be quantified in vivo with high sensitivity and minimal interference from ^1^H-^12^C signals [74].

The advent of hyperpolarization (HP), which can boost the difference in nuclear spin populations between the two spin states of the ^13^C nucleus by several orders of magnitude over that achieved by an applied magnetic field, provides correspondingly huge gains in sensitivity for observation of ^13^C-enriched substrates. However, this advantage can only be realized over a relatively limited time window that is ultimately constrained by the longitudinal relaxation time (T_1_) of the observed ^13^C species. Since ^13^C-^1^H dipolar interactions promote the relaxation of the ^13^C, thereby shortening T_1_, HP studies utilize ^13^C-enriched substrates containing ^13^C that are not directly bound to protons. These nuclei are relaxed via the less efficient chemical shift anisotropy mechanism, resulting in T_1_ values that are several-fold longer compared to proton-bound ^13^C nuclei, but nevertheless rarely exceeding 60 s—and moreover subject to significant reduction by high magnetic fields [75]. Since 99% of nuclear magnetization is lost over an interval of 5 × T_1_, the challenges in rapid administration and in vivo observation of hyperpolarized ^13^C-enriched substrates are reminiscent of those encountered in positron emission tomography (PET) studies of short-lived nuclei such as ^13^N and ^11^C. To date, HP studies of liver metabolism with ^13^C-enriched substrates have been limited to preclinical animal models. While dynamic nuclear polarization (DNP) is the principal method for generating hyperpolarized states in ^13^C-enriched substrates, for certain substrates that can be synthesized via hydrogenation of a ^13^C-enriched precursor, for example [1-^13^C]fumarate from [1-^13^C]acetylene dicarboxylate, polarization via the reductive addition of *para*-hydrogen is both faster and requires less costly equipment compared to DNP [76]. The principal obstacles in the translation of HP to humans have been in ensuring the safety and enabling of rapid delivery of hyperpolarized ^13^C-enriched substrates. Both [1-^13^C]- and [2-^13^C]pyruvate have obtained regulatory approval by the FDA as substrates for hyperpolarized MRI [77]. In a study of patients with prostate cancer, delivery of hyperpolarized [1-^13^C]pyruvate was not associated with any adverse events [78]. Given the diversity of pre-clinical studies of HP ^13^C-enriched substrates in both perfused liver as well as in vivo, it is quite certain that this methodology will be applied to the study of human liver metabolism in the very near future.

### 1.6. In Vivo MRS of Other Nuclei in the Study of Hepatic Metabolism

#### 1.6.1. Deuterium

Deuterium (^2^H) is a quadrupolar nucleus with a spin of 1 and a gyromagnetic ratio that is ~15% that of ^1^H. Its natural abundance is 0.015%, which alongside its limited dispersion (15% that of ^1^H in terms of absolute frequency, Hz) makes it a poor choice for observation of liver metabolites compared to ^31^P or natural abundance ^13^C MRS. However, its low natural abundance also means that ^2^H-enriched substrates, which are up to ~2000 times higher than the background, can be more effectively observed. In addition, ^2^H T_1_ values are much shorter compared to those of ^1^H, ^13^C, or ^31^P allowing more free-induction decays to be collected per unit of time thereby effectively boosting sensitivity. However, for large molecular weight metabolites such as glycogen that exhibit very short spin-spin (T_2_) relaxation times, MR visibility of the ^2^H label may be severely compromised [79]. Since the coupling constants of ^2^H with neighboring ^1^H nuclei are relatively small, ^2^H signals are not substantially degraded by these interactions and can therefore be observed in the absence of broadband ^1^H decoupling. In terms of MR hardware, magnetic field strength is the most important limiting factor in the development of metabolic studies with ^2^H-enriched tracers. De Feyter et al. obtained in vivo ^2^H MR signals at a field of 4 T from human liver following ingestion of a glucose load enriched with [6,6-^2^H_2_]glucose [80]. Under these conditions, there was no resolution of [6,6-^2^H_2_]glucose and [6,6-^2^H_2_]glycogen signals, but given that [6,6-^2^H_2_]glycogen was likely not visible under the parameters used for observation, the signals were presumably those of [6,6-^2^H_2_]glucose.

In cases where the ^2^H label can undergo exchange with ^1^H, for example during conversion of [6,6-^2^H_2_]glucose to [3,3-^2^H_2_]lactate, where the [3,3-^2^H_2_]pyruvate intermediate can exchange its ^2^H with ^1^H body water, the product signal intensity needs to be corrected for this exchange [81]. Also, for ^2^H-enriched substrates whose metabolism involves the cleavage of a ^2^H-^13^C-bond, for example conversion of [2-^2^H]glucose-6-P to fructose-6-P via glucose-6-P isomerase [82] the presence of a significant kinetic isotope effect may substantially alter the rate of tracer metabolism relative to that being traced [81].

The study of liver metabolism can also be undertaken with deuterated water (^2^H_2_O). ^2^H_2_O is inexpensive and can be safely administered to 0.5% body water in humans (~33 times above background) over an indefinite period. The ubiquity of water and metabolite hydrogen exchanges in intermediary metabolic pathways results in the ^2^H-enrichment of a diversity of metabolites including lipids and amino acids. Among other things, the rate of ^2^H enrichment of a given metabolite informs its rate of synthesis and/or turnover. With the advent of very high fields (>10 T), it is likely that hepatic ^2^H signals of metabolites enriched by ^2^H_2_O will be at least partially resolved in vivo for human subjects.

#### 1.6.2. Fluorine

As for ^31^P, fluorine exists in nature as single stable isotope, ^19^F. It has a spin of ½, and its sensitivity is 83% that of ^1^H. It generates sharp NMR signals that cover a wide chemical shift range. Its relaxation properties are similar to that of ^1^H, hence, conventional ^1^H pulse sequences for quantitative measurement of ^1^H metabolite signals can be easily adapted for ^19^F. An adult human has ~2.6 g of fluorine that it is almost entirely distributed as fluoride in teeth and bone. Thus, soft biological tissues have essentially no background ^19^F signal, therefore suppression of superfluous signals is not required and spectrometer sensitivity can be fully exploited.

Essentially all in vivo human studies involving ^19^F have focused on the appearance and/or metabolism of pharmacological agents containing ^19^F as part of their molecular composition. The first report describing the observation of hepatic ^19^F signals in vivo was published by Wolf et al. in 1987 [83]. Three cancer patients were studied with a 1.5 T system following ingestion of the anti-cancer drug 5-fluoro uracil (5-FU). Subsequently, the kinetics of 5-FU appearance in the liver and its bio-transformation to 5-fluoro ureido propionic acid and α-fluoro-β-alanine were documented [84,85,86,87,88]. It was demonstrated that 5-FU was retained longer by tumor tissue compared to the surrounding healthy tissues [84] and that tumor 5-FU levels were positively correlated with the clinical response to treatment [87,89]. In vivo ^19^F MR studies demonstrated that the lifetime of 5-FU within hepatic tumors could be extended by interferon-α [90] and by inhibitors of 5-FU catabolism [91]. In a study performed at 3 T, where different regions of the liver were assayed following ingestion of Capecitabine, a pro-drug that is metabolized to 5-FU via 5′-deoxyfluorocytidine 5′-deoxyfluorouridine, these intermediates, as well as products of 5-FU degradation such α-fluoro-β-alanine and 5-fluoro ureido propionic acid, were detected and quantified. These metabolites were found to be heterogeneously distributed in the liver [92].

Sitafloxacin is a broad-spectrum antibacterial agent that contains a fluorine atom in its chemical structure. Its appearance and washout in the liver was characterized in a group of healthy subjects with a 1.5 T system [93]. These parameters were found to be similar to that measured in plasma using HPLC indicating that this drug was not retained in the liver for any significant time [93]. Niflumic acid is a medication for alleviating pain in muscle and joints and has a trifluoromethyl functional group as part of its structure. A study of healthy male volunteers who ingested a single dose of Niflumic acid was performed at 1.59 T [94]. In addition to the appearance of a ^19^F signal corresponding to niflumic acid, a second signal was observed and was identified as 4′-hydroxy niflumic acid (4-HNA). The washout kinetics of the secondary metabolite was much slower in comparison to that of niflumic acid, and was attributed to the fact that while the parent drug is rapidly cleared via blood and urine, 4-HNA is cleared via the biliary system. To the extent that 4-HNA is recirculated via enterohepatic biliary circulation, its net clearance from the region of observation is slowed down. The authors also acknowledged that the 4-HNA signal might at least in part be originating from the biliary system itself rather than from liver tissue.

## 2. Future Perspectives and Main Conclusions

The two key drivers for the advancement of in vivo MR studies of human liver metabolism are the development of ultra high field clinical MR systems (≥7.0 T) and the availability of hyperpolarized stable-isotope tracers. While each of these technical developments by themselves will undoubtedly advance the state-of-the-art, there is a high degree of synergy when both are combined. This is well illustrated by preclinical studies that have integrated hyperpolarized tracers with in vivo MR observation at mid- to high fields. While high field magnets and hyperpolarization systems come with substantially higher capital and operating costs compared to current clinical MR scanners, they could potentially compete with positron emission tomography (PET) for metabolic imaging applications.

One of the key roles of the liver is the regulation of endogenous glucose production and the control of gluconeogenic flux is a key component of this process. Current methodologies rely on measuring the appearance of a gluconeogenic tracer in plasma glucose. For various technical and theoretical reasons, this measurement is limited to quasi steady-state conditions, such as after overnight fasting or during a glucose clamp. Thus, the transition from fasting to feeding, where hepatic carbohydrate metabolic fluxes must undergo acute rearrangements in order to maintain whole body glucose homeostasis—and is, therefore, the most critical and testing phase for glucoregulation—is little understood. The ability to observe fast real-time alterations in hepatic sugar phosphates and other metabolites following administration of tracers, such as [2-^13^C]dihydroxyacetone [95,96,97], [1-^13^C]pyruvate [98,99,100], and [1-^13^C]gluconolactone [101], promises to be invaluable for unveiling the redirection of hepatic carbohydrate fluxes during the fasted to fed transition. Moreover, the direct observation of hepatic metabolites overcomes another important limitation of gluconeogenic tracer enrichment of blood glucose: the inability to resolve gluconeogenic activity of the liver from that of other tissues such as the kidney and intestine.

For chronic metabolic diseases such as non-alcoholic fatty liver disease (NAFLD) and Type 2 diabetes, there is now renewed focus on the function of hepatocyte mitochondria in these settings. The leakage of electrons from complexes I and III of the electron transport chain results in the generation of reactive oxygen species (ROS). In addition to damaging critical cellular infrastructure such as membrane lipids and DNA, ROS also promote inflammation and can trigger cellular apoptosis and autophagy. Thus, the development of noninvasive hepatic ROS probes, such as hyperpolarized thiourea [102], and markers of hepatic redox state, such as [1-^13^C]alanine and [1-^13^C]lactate [103], will provide a deeper insight on the role and status of hepatic ROS in various physiological and pathophysiological settings. The oxidation of long-chain fatty acids (LCFA) by hepatocyte mitochondria is a critical component in hepatic lipid homeostasis and ketone body generation, and is highly controlled by LCFA uptake via the carnitine shuttle. Defects in hepatic mitochondrial fatty acid oxidation are associated with increased levels of acylcarnitine intermediates [104,105,106]. Therefore, the development of probes for assessing hepatic carnitine metabolism, such as hyperpolarized ^15^N-carnitine [107], can potentially provide information on the status of hepatic LCFA oxidation. Finally, oxidative and anaplerotic pyruvate metabolism—mediated by mitochondrial pyruvate dehydrogenase and pyruvate carboxylase, respectively—is a key node in the hepatic metabolic network. Among other things, it commits pyruvate to either a gluconeogenic or lipogenic fate. The metabolic path of hyperpolarized [1-^13^C]pyruvate can be followed in real time [98], thus providing the potential for a deeper understanding on the role of this critical metabolic control point in various nutritional and disease settings [99,100,108].

After lung cancer, hepatocellular cancer (HCC) is the leading cause of cancer deaths in the world [109] with NAFLD being the most rapidly growing contributor to HCC mortality and morbidity [110]. Noninvasive in situ metabolic profiling of liver tumors will deepen our understanding of tumor physiology and response to therapy and preclincal proof-of-concept studies of hepatic tumors with hyperpolarized substrates are poised to be translated into the clincal setting. The selective uptake and retention of hyperpolarized ethyl [1,3-^13^C_2_]acetoacetate by tumor tissue over healthy hepatocytes [111] provides the basis for tumor metabolic contrast agent imaging and also reveals important differences in carboxyl esterase activities between tumors and healthy tissue that may be exploited for pharmacological targeting. Imaging of tissue pH from hyperpolarized ^13^CO_2_ and bicarbonate delivered in the form of hyperpolarized ethyl acetyl carbonate [112] can potentially delineate tumor necrotic regions which are typically hypoxic and acidic and also increase resistance to therapy. Finally, the local recurrence of HCC following therapy is a frequent and ominous event. Thus, improving our understanding of how tumor cells resist therapy and detection of surviving latent tumor cells is of critical importance in achieving better outcomes. By identifying a characteristic metabolic profile for latent tumor cells via hyperpolarized [1-^13^C]pyruvate that provides the basis for their metabolic imaging, Perkons et al. also demonstrated that metabolic reprogramming is a key component of tumor cell survival [113].

### Main Conclusions

The non-invasive study of human liver metabolism by MRS has been an enduring effort since the initial development of clinical MR scanners. Ongoing technological advances in the design of MR components, such as magnets, RF coils, and gradient systems, are resulting in continuous improvements in the quality and reproducibility of liver metabolite measurements and are also allowing hitherto hidden aspects of liver metabolism to be glimpsed. For some of the most important and prevalent diseases of the current age—notably NAFLD and HCC—it is becoming increasingly clear that their progression is characterized by alterations in hepatic intermediary metabolic fluxes. Importantly, alterations in metabolic flux may not necessarily be accompanied by significant changes in metabolite concentrations. Improvements in sensitivity and resolution of hepatic metabolite enrichments from stable isotope tracers will drive the transition from quantifying metabolite pools, per se, to measuring carbon fluxes through these pools, thereby gaining a much deeper understanding of metabolic alterations in hepatic diseases.

## Figures and Tables

**Table 1:** Selected in vivo ^31^P MRS studies of liver metabolism in human subjects.

Study Description	Main Findings	Field Strength (T)	Reference
Effects of a lipid-rich breakfast meal followed by exercise on hepatic ATP and lipid levels for healthy subjects.	Liver fat increased postprandially and continued to increase during exercise. Liver ATP did not change from fasting to postprandial state, but significantly decreased after exercise.	3.0	[51]
Effect of a oral fructose challenge on hepatic ATP reserves in healthy subjects. Baseline liver glycogen was also measured by ^13^C NMR	Hepatic ATP levels dropped by ~20% from baseline and reached a minimum value 50 min after the load. The time to reach minimum ATP levels was inversely correlated with subject BMI. ATP recovery rate was inversely correlated with baseline glycogen levels.	3.0	[52]
Effects of acute fructose ingestion with and without an accompanying load of ethanol on liver P-metabolite dynamics in healthy subjects.	Over a 40 min interval post load, P-metabolites were measured with 5 min time resolution. While ethanol had no effects on rates of phosphomonester (PME) formation and ATP depletion resulting from fructose metabolism, it significantly slowed down the rate of PME degradation.	1.5	[53]
Characterization of P-metabolites and ATP fluxes and correlation with lipid levels determined by ^1^H MR and biopsy evaluation in subjects with NAFLD and NASH	Several PME and PDE ^31^P signals were resolved and quantified as well as those from NADPH and UDPG. Significant differences in relative abundances of PME phosphoethanolamine (PE) and ATP between NAFLD and NASH. Significantly lower rates of ATP synthesis fluxes in NASH compared to NAFLD subjects [33]. In another ^31^P MRS study performed at 3 T, levels of NADPH, a marker of inflammation and fibrosis, were elevated in NASH patients compared to healthy controls [54].	7.0, 3.0 (^31^P) 3.0 (^1^H)	[33,54]
Characterization of PME profile in fasted subjects with compensated and decompensated cirrhosis following infusion with a gluconeogenic substrate—L-alanine.	At baseline, PME levels of both compensated and decompensated cirrhotic subjects were elevated compared to healthy controls. After L-alanine infusion, PME levels of healthy controls were significantly increased, consistent with gluconeogenic activity. This increase was significantly smaller for patients with compensated cirrhosis and was absent in patients with decompensated cirrhosis.	1.5	[55]
Characterization of P-metabolites in pediatric liver transplant patients with different outcomes of graft function	Patients with impaired graft function had elevated PME/total phosphate compared to those with good graft function and to healthy controls.	1.5	[56]
Effects of intravenous ATP infusion for 22–24 h on liver energy status in advanced lung cancer patients.	Liver ATP levels were significantly increased following ATP infusion to levels that were similar to those of healthy subjects. This effect was greatest for patients that were undergoing weight loss and who had the lowest baseline ATP liver levels	1.5	[57]
